# Factors Influencing Self-Management Behaviors among Hemodialysis Patients

**DOI:** 10.3390/jpm12111816

**Published:** 2022-11-02

**Authors:** Li-Ching Ma, Yueh-Min Liu, Yen-Chung Lin, Chia-Te Liao, Kuo-Chin Hung, Remy Chen, Kuo-Cheng Lu, Kuei-Fang Ho, Cai-Mei Zheng

**Affiliations:** 1 Department of Nursing, Hemodialysis Center, Cardinal-Tien Hospital, New Taipei City 231, Taiwan; 2 Department of Nursing, Ching Kuo Institute of Management and Health No. 336, Fu-Sing Rd., Jhongshan Dis., Keelung 203, Taiwan; 3 Division of Nephrology, Department of Internal Medicine, Taipei Medical University Hospital, Taipei 110, Taiwan; 4 Department of Internal Medicine, School of Medicine, College of Medicine, Taipei Medical University, Taipei 110, Taiwan; 5 TMU Research Center of Urology and Kidney (TMU-RCUK), Taipei Medical University, Taipei 110, Taiwan; 6 Division of Nephrology, Department of Internal Medicine, Shuang Ho Hospital, Taipei Medical University, New Taipei City 235041, Taiwan; 7 Division of Nephrology, Department of Medicine, Min-Sheng General Hospital, Taoyuan 330, Taiwan; 8 Division of Nephrology, Kimura Hospital, Tokyo 116-0001, Japan; 9 Division of Nephrology, School of Medicine, Taipei Tzu Chi Hospital, Buddhist Tzu Chi Medical Foundation, Buddhist Tzu Chi University, Hualien 97004, Taiwan; 10 School of Medicine, Fu-Jen Catholic University, New Taipei City 242, Taiwan; 11 School of Nursing, College of Nursing, Taipei Medical University, Taipei 110, Taiwan

**Keywords:** hemodialysis patients, self-management behavior, depression, social support, comorbid conditions

## Abstract

Aim: To investigate the factors affecting hemodialysis patients’ self-management ability at a dialysis center in Taiwan. Background: Taiwan has the highest incidence and prevalence of end-stage kidney disease (ESKD) in the world. Over 90% of patients with ESKD receiving hemodialysis (HD) and self-management behaviors are critical among these patients. Failure to adhere to self-managed care increases the cost of medical care and the risk of morbidity and mortality. Methods: In this cross-sectional study, a total of 150 HD patients were observed for their self-management behaviors and the factors influencing these behaviors including education level, comorbid conditions, biochemical analysis, depression, and social support, etc., were analyzed. Results: Self-management behaviors in HD patients were significantly impaired in the presence of diabetes mellitus, hypertension, anemia, hypoalbuminemia, and depression. The major predictor of patients’ self-management was depression, explaining 14.8% of the total variance. Further addition of social support, hypertension, and diabetes mellitus into the regression model increased the total explained variance to 28.6%. Of the various domains of self-management, the partnership domain received the highest score, whereas emotional processing received the lowest score. Conclusions: This study found the important factors influencing self-management behaviors; through this acknowledgement and early correction of these factors, we hope to improve HD patients’ individual life quality and further decrease their morbidity and mortality.

## 1. Introduction

End-stage kidney disease (ESKD) and dialysis is the most important chronic disease in Taiwan [1]. According to Taiwan’s National Health Insurance Administration (NHIA) [2], care of these patients requires a massive and fast-growing medical expense and places a major burden on health insurance. In addition to long-term dialysis, they also have health problems related to underlying chronic comorbidities. The most common comorbidity is hypertension, accounting for 71.67%, followed by diabetes mellitus at 56.67% [3]. More than 90% of dialysis patients have anemia and experience dyspnea, depression, and headaches, etc. Furthermore, the relative risk of mortality is 1.33 times higher in those with hematocrit (less than 27%) as compared to those with hematocrit (30–33%) [4]. Malnutrition is also a common issue that leads to adverse effects on patients’ health and quality of life, with one-third of patients demonstrating mild or moderate malnutrition and 6–8% demonstrating severe malnutrition [5].

Dialysis patients experience negative feelings toward the life changes brought on by their dialysis treatment; among patients receiving hemodialysis (HD), the prevalence of depression is 20–30% and depression becomes more severe with the progression of the illness, increasing the risk of hospitalization and death [6]. Active participation in home self-monitoring and self-management of comorbid conditions, including regular blood glucose and blood pressure measuring, etc., become critical roles in these dialysis patients’ lives to improve their life quality and quantity after dialysis therapy. Each patient is their own primary disease controller and must learn to shift from their initial dependence on the care provided by their medical team and caregivers to proactive learning of self-management. Furthermore, social support for dialysis patients can provide them with a social network according to their individual need, improving their self-worth, preventing them from pathological symptoms brought on by stress, and providing positive influences on their physical health, mental health, and social functions [7]. Therefore, identifying the factors influencing HD patients’ self-management behaviors in advance can help the adoption of prompt, appropriate interventive measures before the development of visible comorbidities. This, in turn, prevents admission rates and reduces the risk of comorbidities, thereby improving the quality of life for these HD patients.

Wang et al. [8] defined self-management behaviors in chronic diseases as the patient’s proactive engagement in health care activities to learn to solve problems, control their disease, and adjust their way of living to coexist with their chronic disease in everyday life; effective self-management includes the ability to monitor the individual’s own condition and emotions to maintain a satisfying quality of life and reduce medical costs [8]. Chan et al. [9] also demonstrated a case management model that brings clinical professionals from different disciplines together to provide comprehensive and continuous care, which can effectively increase ESKD patients’ understanding of their disease and increase their satisfaction with their medical care [9]. Since ESKD is irreversible and patients need lifelong dialysis therapy, their self-management behaviors play a critical role in determining their life quality and quantity. In this study, we explored the factors influencing the self-management behaviors among ESKD patients under regular HD at a Northern Taiwan teaching hospital hemodialysis center.

## 2. Methods

### 2.1. Study Design

A cross-sectional observational study was conducted at a Northern Taiwan teaching hospital hemodialysis (HD) center from July 2019 to April 2020. We recruited a total of 150 HD patients with the following inclusion criteria: (1) diagnosed with end-stage kidney disease and under maintenance HD for more than 3 months, (2) age between 20 and 80 years and able to provide informed consent, and (3) ability to converse well in Mandarin or Taiwanese.

### 2.2. Sample and Setting

After obtaining informed consent, the participants were asked to fill out a set of questionnaires. Each questionnaire took approximately 20 min to complete. In total, 160 patients contributed completed questionnaires and 10 patients were excluded due to poor health condition, resulting in 150 valid questionnaires. G-Power, developed by Erdfelder et al., was used to calculate the sample size in this study [10]. The α value was set to 0.05, power set to 0.80, and the effect size set to 0.25. The predicted sample size was 120 people but the actual number of participants in this study was 150; therefore, the power reached 0.87.

The study data were encoded, archived, and analyzed using SPSS version 22.0, SPSS Inc., Chicago, IL, USA. Descriptive statistics are presented as frequency and percentages or mean ± standard deviations. Inferential statistics were analyzed using t tests, one-way ANOVA, and stepwise regression.

### 2.3. Research Tools

A structured questionnaire included questions on demographic traits, test indicators, and three instruments. The questionnaire contents are introduced as follows:

1. Demographic traits:

Demographic traits include the participants’ sex, age, education level, and other comorbid conditions, such as hypertension and diabetes mellitus.

2. Defining and measuring biochemical parameters:

The biochemical parameters of participants, including hemoglobin and albumin variables, were recorded. Kidney Disease: Improving Global Outcomes (KDIGO) anemia work group [11] recommended that the normal hemoglobin for HD patients should be ≥10 g/dL. In this study, patients with hemoglobin less than 10 g/dL were considered anemic. Albumin is the most common physiological indicator of malnutrition and was measured using bromocresol purple (BCP). According to the National Health Insurance Administration [2], albumin (BCP) levels of ≥3.5 g/dL indicate appropriate nutrition. Clinical studies revealed that albumin levels <3.8 g/dL extend hospital stays and increase mortality rates [12]. Among patients receiving HD treatments worldwide, high albumin levels can reduce the rate of cardiac death. As such, patients are recommended to ingest protein and maintain albumin levels of ≥4.0 g/dL [13]. In this study, albumin levels <4.0 g/dL was defined as hypoalbuminemia.

3. Beck Depression Inventory (BDI):

The Beck Depression Inventory (BDI) [14] is often used to measure levels of depression. In Taiwan, this instrument was translated into Chinese in 1995 [3] and was exclusively published in Taiwan by the Chinese Behavioral Science Corporation, with the authorization of NCS Pearson [15]. The inventory includes 21 items and each item is rated from 0 = no depression to 3 = very depressed. Total scores are between 0 and 63, with higher scores indicating higher levels of depression. BDI scores of 0–13 were interpreted as normal and a score of ≥14 indicated depression. In a Taiwanese study examining the depression experienced by 60 end stage kidney disease (ESKD) patients receiving HD, the Cronbach’s α of BDI was determined to be 0.93 [16].

4. Social support instrument:

The 17-item instrument for measuring social support among HD patients that was used in this study was well-developed [17]. The instrument comprises two parts: nine items related to support from friends and family and eight items related to support from medical staff. The total score of the instrument is 17–68, with higher scores indicating greater social support. The Cronbach’s α of the instrument’s internal reliability was 0.91, demonstrating that the instrument was fairly robust [18].

5. Hemodialysis Self-Management Instrument (HD-SMI):

The instrument measuring HD patients’ self-management behaviors [19] has been developed to evaluate the self-management behaviors of patients. This instrument measures a patient’s ability to manage their own disease and was tested on 196 HD patients. This instrument contains four domains and 20 items that describe disease self-management behaviors in the past 7 days. Each item is graded on a 4-point scale: 0 for never or rarely (no more than one day each week), 1 for sporadically (1 to 2 days each week), 2 for occasionally (3–4 days each week), and 3 for frequently (5–7 days each week) [19]. The total score range was 0–60 and higher scores indicate more favorable self-management behaviors. The Cronbach’s α coefficient denoting the internal inconsistency of the instrument was 0.87, indicating that it is fairly consistent [19].

### 2.4. Data Collection Process and Ethical Consideration

This study was approved by Institutional Review Board of Cardinal Tien Hospital (CTH-108-3-5-015; 12 July 2019). Initially, a pilot study was performed with 20 patients with chronic kidney disease (CKD). The researchers collected data through face-to-face interviews with the participants. Each session was approximately 20 min and no difficulties were encountered. Soon afterwards, the formal study was conducted. Written informed consent was obtained from all participants, who were notified that they could withdraw from the study (i.e., cease filling the questionnaire) at any time without affecting their treatment. The test items were determined using their medical records. In total, 160 questionnaires were distributed; 10 participants withdrew because of poor health, leaving 150 valid questionnaires.

## 3. Results

### 3.1. Demographics, Disease Characteristics, Social Support, and Depression among HD Patients

Of the 150 patients receiving HD treatments, 78 were men (52%) and 72 were women (48%). The participant mean age was 67.2 ± 13.3 years. Most participants had high (vocational) school or higher education, comprising 88 participants (58%), followed by junior high school with 48 participants (32%). A total of 85 participants (56.7%) had diabetes mellitus and 87 participants (58%) had hypertension. The mean hemoglobin level was 10.3 ± 1.5 g/dL and 57 participants (38%) had anemia. The mean albumin level was 3.8 ± 0.39 g/dL and 103 participants (68.7%) had hypoalbuminemia. A total of 36 participants (24%) had depression and 81 participants (54%) received low social support. Table 1 summarizes the participants’ baseline demographic information.

The analysis of the HD patients and their self-management behaviors in Table 1 indicates that patients with a higher education and higher hemoglobin and albumin levels revealed significantly better self-management behaviors. Those with diabetes mellitus, hypertension, or depression demonstrated less favorable self-management behaviors. No significant differences were noted for gender and social support.

### 3.2. Self-Management Behaviors among HD Patients

The HD patients’ overall self-management scores ranged from 0 to 60 (mean: 25.77 ± 11.8). The score for each domain was divided by the number of items in that domain to determine the mean item score. The partnerships domain consisted of four items and had a mean item score of 1.5 points, the highest among the domains. The mean item score in the emotional processing domain was 1.02 points, the lowest among the domains. Table 2 provides information on other domain scores.

### 3.3. Factors in Self-Management Behaviors among the HD Patients

Pearson correlation coefficient analysis of HD patients’ self-management behaviors revealed a significant correlation between the education level and self-management scores (r = 0.215, *p* = 0.008). Diabetes mellitus and hypertension had a significant negative correlation with self-management scores (r = −0.222, *p* = 0.006 and r = −0.289, *p* = 0.001, respectively). A significant positive correlation was noted between hemoglobin and albumin levels with self-management scores (r = 0.182, *p* = 0.025 and r = 0.251, *p* = 0.002, respectively). A significant negative correlation was present between the level of depression and self-management (r = −0.389, *p* = 0.001), whereas a significant positive correlation was present between social support and self-management (r = 0.230, *p* = 0.005) (Table 3).

### 3.4. Major Predictors of Self-Management Behaviors

The significant variables, namely the total depression score, total social support score, presence of diabetes mellitus, and hypertension identified in the bivariate analysis were adopted as the independent variables used to perform stepwise regression. The variables explained 28.6% of the total variance, with depression as the major predictor explaining 14.8% of the total variance (Table 4).

## 4. Discussion

Our study provides new insight into factors influencing self-management behaviors among dialysis patients taking consideration for various aspects. The mean age of our study patients is 67.2 years, where 58.87% received a high (vocational) school-level education degree or above, and their mean self-management score was 25.77. A significant and positive correlation was observed between self-management and education. This is consistent with the results of previous studies that found that higher education improves self-management and cognitive activities [20,21]. Common chronic diseases experienced by patients receiving HD treatments include hypertension and diabetes mellitus. These chronic diseases were observed to have a significant and negative correlation with self-management. Lee et al. [3] also noticed that patients with hypertension and diabetes mellitus receiving HD treatments exhibited poor self-management. Self-management behaviors are important in these patients since regular home blood pressure and glucose monitoring improve their prognosis and compliance. We cannot explain why HD patients with such comorbid conditions have lower self-management scores; however, their poor self-management might be important cause of chronic kidney disease (CKD) deterioration to ESKD.

In biochemical analysis, a significant correlation was noted between hemoglobin levels and self-management scores; higher levels of hemoglobin were associated with higher self-management scores. In dialysis centers, HD patients’ hemoglobin levels were regularly tested and erythropoietin or iron therapy were adjusted as needed to maintain hemoglobin levels of ≥10 g/dL. Anemia presented with various symptoms including fatigue, dyspnea, dizziness, insomnia, left ventricle hypertrophy, etc. The need for frequent blood transfusions and hospitalizations further increases their morbidity and impairs their ability for self-management. Increased levels of hemoglobin result in higher physical strength and more favorable self-management [22]. This finding is consistent with that of Kang et al. [23], who reported a significant and positive correlation between hemoglobin levels and self-management scores. On the other hand, a significant correlation was observed between albumin levels and self-management scores. Higher levels of albumin were associated with higher self-management scores. A previous study on incident dialysis patients [24] also revealed similar findings, where patients were able to adhere to dietary self-management after receiving nutritional consultations and higher albumin levels [24]. On the other hand, a non-significant correlation between albumin levels and self-management scores was noted among 75 participants who receive 8 weeks of remote health education [5]. This suggests that self-management scores might vary with methods of health education and importance of dietary education [5].

In this study, chronic HD patients exhibited moderate levels of self-management behaviors, with the partnership domain having the highest mean score and emotional processing having the lowest. The partnership role in self-management behaviors refers to the patient and medical staff discussing the various aspects of the patient’s HD treatment, such as fluid control and dry weight control, fluid amount to be removed during every dialysis session, HD flow rate, and positions of the arteriovenous fistula injections. The patient received two to three HD treatment sessions each week and each session lasted 4 h. During each session, the nurses discussed vascular access, hydration, diet, and other self-care behaviors with the patient and encouraged the patient to practice self-management at home; therefore, these participants scored highest in the partnership domain. This result is consistent with Lee et al. [3], who also pointed out the importance of the partnership role. In contrast, the emotional processing domain had the lowest scores. HD treatment exerts a notable effect on patients’ psychological adjustments. Although such treatment can alleviate relevant symptoms and extend patients’ lifespan, their physical strength and activities of daily life are affected by the accompanying changes in their way of living and dietary habits, as well as the physical discomfort induced by the treatment. Lin et al. [25] interviewed dialysis patients, who stated that society has a specific and negative perception of renal diseases; consequently, they also have to face the problems of self-blame and depression while coping with their diseases. A significant and negative correlation was observed between the depression level and self-management among HD patients. We revealed that depression was the strongest predictor of self-management, explaining 14.8% of the total variance. In other words, severe depression could lead to unfavorable self-management. Kim et al. [26] also found that depression is a major predictor of self-management, with a total explained variance of 24.9%. Depression reduces HD patients’ self-management ability. Patients need to receive two to three sessions of dialysis treatment every week and therefore are prone to experience fatigue, insomnia, poor appetite, and other symptoms of physical distress, which in turn increase their level of depression and thereby affect their self-management. Previous studies reported that depression has a significant and negative correlation with self-management behaviors [27]. Our previous study also revealed a close relation between pro-inflammatory cytokines and poor nutritional status among dialysis patients [28]. Furthermore, similar results are present in self-management among patients with kidney transplants and those with chronic kidney diseases; the total variance explained by depression in these patients was 14.4% [29]. Therefore, helping these patients to better understand the depression and related behaviors, earlier detection, and receiving treatment in advance improve patients’ self-management behaviors.

Social support was the second major predictor for self-management and was positively correlated with self-management behaviors. Previous studies [26,30] also concluded that social support and self-care were positively correlated. In this study, social support was defined as support from family members and medical staff. In addition to providing care in various aspects of living at home, including diet, clothing, accommodations, and transportation, most family members’ support was also important in these patients. Furthermore, patients receiving HD treatments must undergo 4 h of treatment at the hospital, two to three times each week, and their relationship with the medical staff is similar to that with family members; medical staff inquire after the patient’s condition and manage the patient’s discomfort at any time, allowing the patient to feel greater professional support and improving the patient’s self-management. The participants agreed that the medical staff were professionals and that their instruction should therefore be followed. The participants also acknowledged the support given by medical staff. Effective social support may have increased patients’ willingness to understand and reinforce their self-management.

This study still has several limitations; a great limitation is the cross-sectional research design where we could only measure the self-management of HD patients during the study period. This type of research design precludes the long-term or continuous assessment of patients’ self-management at different stages. Furthermore, since this study only recruited participants from a single hospital and the study results are not generalizable to all HD patients. However, future researchers can incorporate variables associated with personality traits, stress, fatigue, and physical symptoms and distress to further explore approaches to enhancing self-management behaviors among HD patients. Longitudinal study designs can be adopted to conduct long-term and continuous assessments of patients’ self-management at different stages of their treatment.

Overall, our study indicated that HD patients with lower levels of depression and greater social support and without high blood pressure or diabetes mellitus demonstrated more favorable self-management behaviors. A negative correlation was observed between psychological distress and self-management; severe psychological distress was associated with poorer self-management abilities. Targeting the factors influencing self-management behaviors are critically important for HD patients to improve their quality of life and further decrease their morbidity and mortality.

## Figures and Tables

**Table 1:** Baseline characteristics of study patients according to their self-management behaviors (*N* = 150).

Variables	*N*	%	Self-Management Behaviors	T/F	*p*
			Mean ± SD		
Sex				1.854	0.175
Men	78	52%	24.68 ± 11.33		
Women	72	48%	26.94 ± 12.24		
Education level				3.561 *	0.031
Illiterate	14	9.3%	20.21 ± 11.97		
Elementary and junior high school	48	32.0%	23.79 ± 11.84		
High (vocational) school or higher	88	58.7%	27.73 ± 11.40		
Diabetes mellitus				2.774 **	0.006
Yes	85	56.7%	23.48 ± 10.98		
No	65	43.3%	28.75 ± 12.23		
Hypertension				3.677 ***	<0.001
Yes	87	58.0%	22.87 ± 11.19		
No	63	42.0%	29.76 ± 11.51		
Hemoglobin				2.522 *	0.013
<10 g/dL	57	38.0%	22.72 ± 11.88		
≥10 g/dL	93	62.0%	27.63 ± 11.40		
Albumin				2.617 **	0.01
<4.0 g/dL	103	68.7%	24.10 ± 11.66		
≥4.0 g/dL	47	31.3%	29.43 ± 11.35		
Depression				4.391 ***	<0.001
Depression (14+)	36	24.0%	18.67 ± 12.23		
No depression (0–13)	114	76.0%	28.01 ± 10.76		
Social support				2.168 *	0.032
Low (17–51 points)	81	54.0%	23.86 ± 11.48		
High (52–68 points)	69	46.0%	28.00 ± 11.84		

* *p* < 0.05, ** *p* < 0.01, *** *p* < 0.001.

**Table 2:** Self-management scale and its domains (*N* = 150).

Item	Number of Items	Score Range	Lowest Score	Highest Score	Mean Score	Standard Deviation	Mean Score per Item	Order
Overall self-management	20	0–60	2	48	25.77	11.8	1.29	
Partnerships	4	0–12	0	12	6.00	3.5	1.50	1
Performing self-care activities	7	0–21	1	19	9.07	4.4	1.29	3
Problem-solving	5	0–15	0	15	6.77	3.7	1.35	2
Emotional processing	4	0–12	0	12	4.09	2.7	1.02	4

**Table 3:** Bivariate analysis of HD patients’ self-management (*N* = 150).

	Self-Management	
Variables	r	*p*
Education level	0.215 **	0.008
Diabetes mellitus	−0.222 **	0.006
Hypertension	−0.289 ***	0.001
Hemoglobin level	0.182 *	0.025
Albumin level	0.251 **	0.002
Depression	−0.389 ***	0.001
Social support	0.230 **	0.005

* *p* < 0.05, ** *p* < 0.01, *** *p* < 0.001.

**Table 4:** Stepwise regression analysis of self-management variables (*N* = 150).

Variables	Regression Coefficient B Value	Standardized *β* Value	R^2^	Adjusted R^2^	*t* Value	*p*
Total depression score	−0.66	−0.392	0.154	0.148	−5.168 ***	<0.001
Total social support score	0.495	0.280	0.231	0.221	3.86 ***	<0.001
Presence of Hypertension	−4.845	−0.203	0.270	0.255	−2.782 **	0.006
Presence of Diabetes Mellitus	−4.498	−0.190	0.305	0.286	−2.702 **	0.008

** *p* < 0.01, *** *p* < 0.001.

## References

[1] Saran R., Robinson B., Abbott K.C., Agodoa L.Y., Bhave N., Bragg-Gresham J., Shahinian V. (2018). Us renal data system 2017 annual data report: Epidemiology of kidney disease in the united states. Am. J. Kidney Dis..

[2] National Health Insurance Administration Actual and Valid Proof of Major Illness and Injuries under the National Health Insurance. https://www.nhi.gov.tw/Content_List.aspx?n=D529CAC4D8F8E77B&topn=23C660CAACAA159D.

[3] Lee M.C., Lu K.C., Wu S.F.V., Hsieh H.L., Liu Y.M. (2016). Effectiveness of self-management program on improving self-efficacy and depression in patients with hemodialysis. Taipei City Med. J..

[4] Lee C.Y., Yeh H.C. (2018). Diagnosis and treatment of anemia in patients with chronic kidney disease. Kidney Dial..

[5] Pack S., Lee J. (2021). Randomised controlled trial of a smartphone application-based dietary self-management program on haemodialysis patients. J. Clin. Nurs..

[6] Huang C.W., Wee P.H., Low L.L., Koong Y.L.A., Htay H., Fan Q., Foo W.Y.M., Seng J.J.B. (2021). Prevalence and risk factors for elevated anxiety symptoms and anxiety disorders in chronic kidney disease: A systematic review and meta-analysis. Gen. Hosp. Psychiatry.

[7] Stevenson J., Tong A., Campbell K.L., Craig J.C., Lee V.W. (2018). Perspectives of healthcare providers on the nutritional management of patients on haemodialysis in australia: An interview study. BMJ Open.

[8] Wang S.L., Kung L.F., Chen T.H., Hsiao S.M., Hsiao P.N., Chiou C.J. (2016). Construction and validation of a chronic kidney disease self-care scale. Hu Li Za Zhi.

[9] Chan I.T., Yang H.L. (2018). An exploration of the development of chronic disease self-management in Macao. Macau J. Nurs..

[10] Erdfelder E., Faul F., Buchner A. (1996). Gpower: A general power analysis program. Behav. Res. Methods Instrum. Comput..

[11] McMurray J., Parfrey P., Adamson J.W., Aljama P., Berns J.S., Bohlius J., Weiss G. (2012). Kidney disease: Improving global outcomes (KDIGO) anemia work group. KDIGO clinical practice guideline for anemia in chronic kidney disease. Kidney Int. Suppl..

[12] Ikizler T.A., Burrowes J.D., Byham-Gray L.D., Campbell K.L., Carrero J.J., Chan W., Cuppari L. (2020). KDOQI clinical practice guideline for nutrition in ckd: 2020 update. Am. J. Kidney Dis..

[13] Ma L., Zhao S. (2017). Risk factors for mortality in patients undergoing hemodialysis: A systematic review and meta-analysis. Int. J. Cardiol..

[14] Beck A.T., Epstein N., Brown G., Steer R.A. (1988). An inventory for measuring clinical anxiety: Psychometric properties. J. Consult. Clin. Psychol..

[15] Lu M.L., Che H.H., Chang S.W., Shen W.W. (2002). Reliability and validity of the Chinese version of the Beck Depression Inventory-II. Taiwan. J. Psychiatry.

[16] Hsu S.Y., Huang H.S. (2019). Improving depression, hope, and quality of life in dialysis patients using health promotion education groups. Hu Li Za Zhi.

[17] Lin C.C., Ko N.Y., Tsai L.C., Chen C.H. (1995). Assessing the effect of health belief, knowledge, and social support on compliance behaviors in chronic hemodialysis patients. Kaohsiung J. Med. Sci..

[18] Chen P.C., Lu K.C., Lee P.H., Miao N.F. (2020). Factors associated with social support, self-care behavior, and the quality of life among hemodialysis patients. New Taipei J. Nurs..

[19] Song Y.C., Lin C.C. (2009). The development and testing of a new hemodialysis self-management instrument (hd-smi). J. Nurs. Healthc. Res..

[20] Gesualdo G.D., Duarte J.G., Zazzetta M.S., Kusumota L., Say K.G., Pavarini S.C.I., Orlandi F.S. (2017). Cognitive impairment of patients with chronic renal disease on hemodialysis and its relationship with sociodemographic and clinical characteristics. Dement. Neuropsychol..

[21] Gela D., Mengistu D. (2018). Self-management and associated factors among patients with end-stage renal disease undergoing hemodialysis at health facilities in addis ababa, ethiopia. Int. J. Nephrol. Renovasc. Dis..

[22] Karaboyas A., Morgenstern H., Fleischer N.L., Vanholder R.C., Dhalwani N.N., Schaeffner E., Robinson B.M. (2020). Inflammation and erythropoiesis-stimulating agent response in hemodialysis patients: A self-matched longitudinal study of anemia management in the dialysis outcomes and practice patterns study (dopps). Kidney Med..

[23] Kang B., Lee Y., Sok S. (2020). Influence of Illness Perception and Physiological Indicators on Self Management of Hemodialysis Patients. J. Korea Contents Assoc..

[24] Liu H.Y., Chang S.H. (2019). Changes in the malnutrition-inflammation score in patients new to hemodialysis after nutrition counseling. Taiwan J. Dietetics..

[25] Lin C.C., Lee B.O., Hicks F.D. (2005). The phenomenology of deciding about hemodialysis among taiwanese. West J. Nurs. Res..

[26] Kim B., Kim J. (2019). Influence of uncertainty, depression, and social support on self-care compliance in hemodialysis patients. Ther. Clin. Risk Manag..

[27] Natashia D., Yen M., Chen H.M., Fetzer S.J. (2019). Self-management behaviors in relation to psychological factors and interdialytic weight gain among patients undergoing hemodialysis in indonesia. J. Nurs. Scholarsh..

[28] Hung K.C., Wu C.C., Chen H.S., Ma W.Y., Tseng C.F., Yang L.K., Lu K.C. (2011). Serum il-6, albumin and co-morbidities are closely correlated with symptoms of depression in patients on maintenance haemodialysis. Nephrol. Dial. Transplant..

[29] Lin M.Y., Liu M.F., Hsu L.F., Tsai P.S. (2017). Effects of self-management on chronic kidney disease: A meta-analysis. Int. J. Nurs. Stud..

[30] Untas A., Thumma J., Rascle N., Rayner H., Mapes D., Lopes A.A., Combe C. (2011). The associations of social support and other psychosocial factors with mortality and quality of life in the dialysis outcomes and practice patterns study. Clin. J. Am. Soc. Nephrol..

